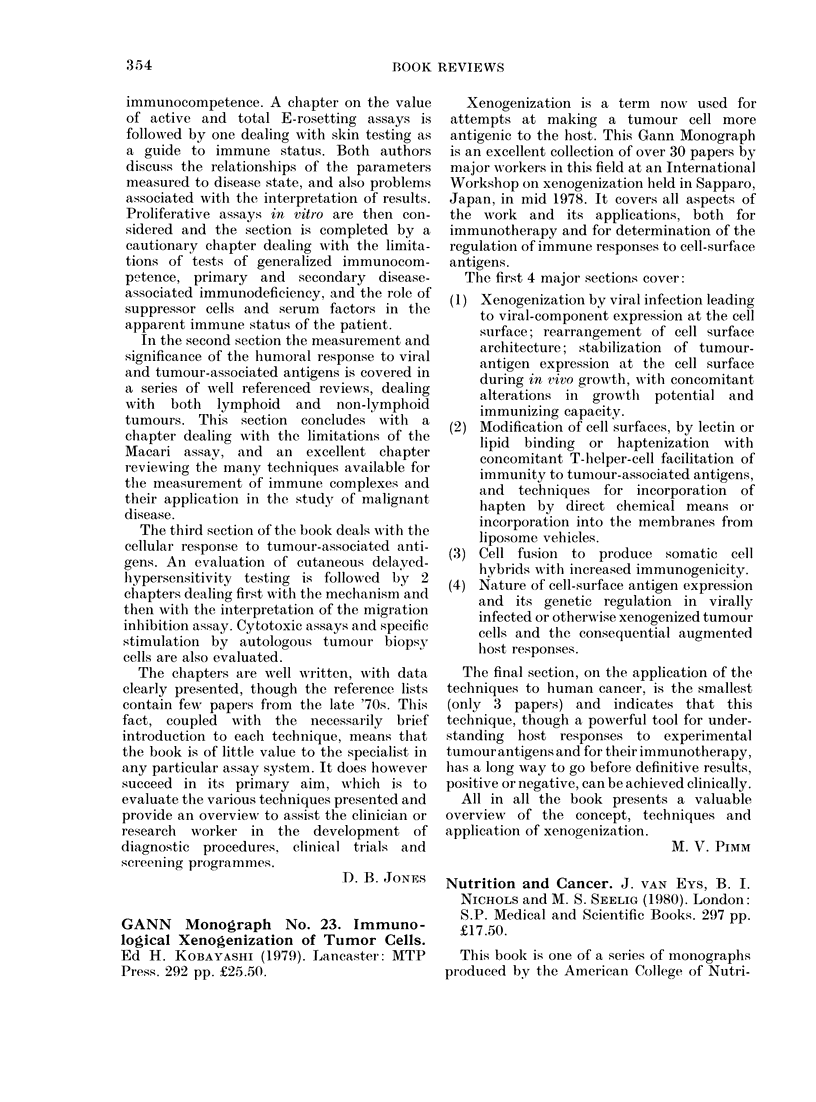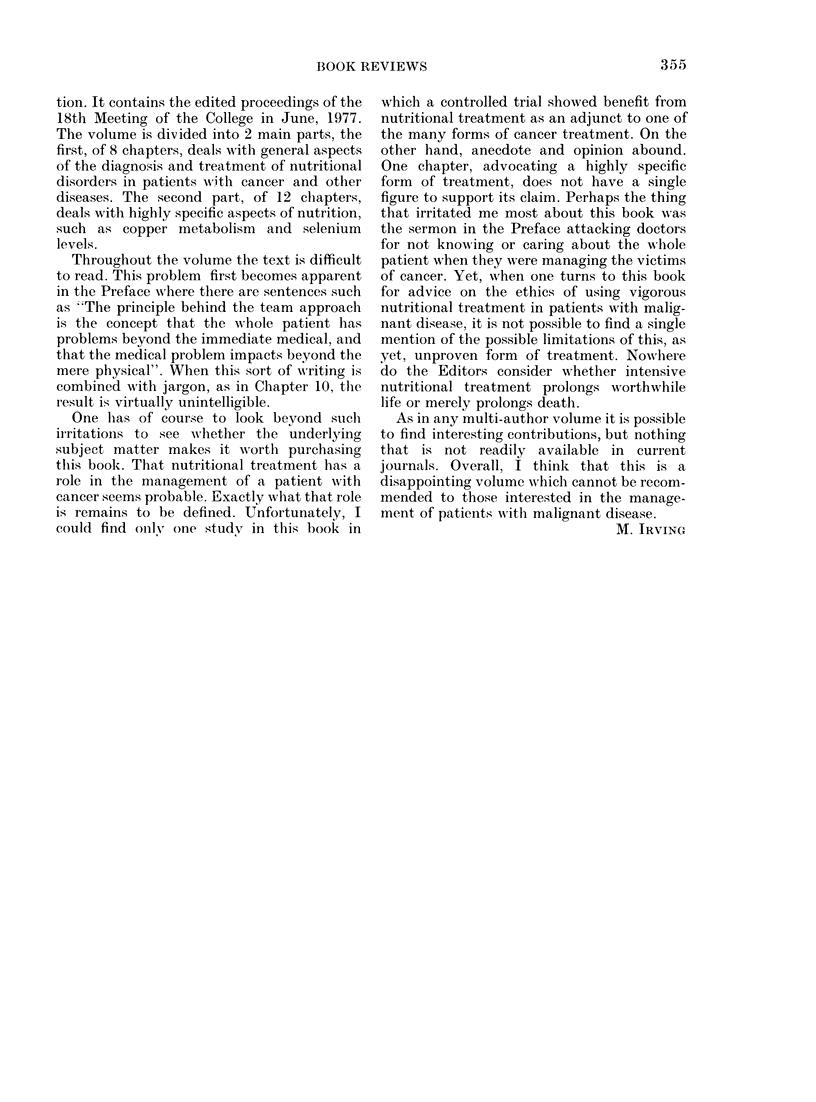# Nutrition and Cancer

**Published:** 1980-08

**Authors:** M. Irving


**Nutrition and Cancer.** J. van Eys, B. I. Nichols and M. S. Seelig (1980). London: S.P. Medical and Scientific Books. 297 pp. £17.50.

This book is one of a series of monographs produced by the American College of Nutrition. It contains the edited proceedings of the 18th Meeting of the College in June, 1977. The volume is divided into 2 main parts, the first, of 8 chapters, deals with general aspects of the diagnosis and treatment of nutritional disorders in patients with cancer and other diseases. The second part, of 12 chapters, deals with highly specific aspects of nutrition, such as copper metabolism and selenium levels.

Throughout the volume the text is difficult to read. This problem first becomes apparent in the Preface where there are sentences such as "The principle behind the team approach is the concept that the whole patient has problems beyond the immediate medical, and that the medical problem impacts beyond the mere physical". When this sort of writing is combined with jargon, as in Chapter 10, the result is virtually unintelligible.

One has of course to look beyond such irritations to see whether the underlying subject matter makes it worth purchasing this book. That nutritional treatment has a role in the management of a patient with cancer seems probable. Exactly what that role is remains to be defined. Unfortunately, I could find only one study in this book in which a controlled trial showed benefit from nutritional treatment as an adjunct to one of the many forms of cancer treatment. On the other hand, anecdote and opinion abound. One chapter, advocating a highly specific form of treatment, does not have a single figure to support its claim. Perhaps the thing that irritated me most about this book was the sermon in the Preface attacking doctors for not knowing or caring about the whole patient when they were managing the victims of cancer. Yet, when one turns to this book for advice on the ethics of using vigorous nutritional treatment in patients with malignant disease, it is not possible to find a single mention of the possible limitations of this, as yet, unproven form of treatment. Nowhere do the Editors consider whether intensive nutritional treatment prolongs worthwhile life or merely prolongs death.

As in any multi-author volume it is possible to find interesting contributions, but nothing that is not readily available in current journals. Overall, I think that this is a disappointing volume which cannot be recommended to those interested in the management of patients with malignant disease.

M. Irving